# In-Situ Stretching Patterned Graphene Nanoribbons in the Transmission Electron Microscope

**DOI:** 10.1038/s41598-017-00227-3

**Published:** 2017-03-16

**Authors:** Zhongquan Liao, Leonardo Medrano Sandonas, Tao Zhang, Martin Gall, Arezoo Dianat, Rafael Gutierrez, Uwe Mühle, Jürgen Gluch, Rainer Jordan, Gianaurelio Cuniberti, Ehrenfried Zschech

**Affiliations:** 1 Fraunhofer Institute for Ceramic Technologies and Systems (IKTS), 01109 Dresden, Germany; 2 Institute for Materials Science and Max Bergmann Center of Biomaterials, Technische Universität Dresden, 01069 Dresden, Germany; 3 Center for Advancing Electronics Dresden (cfaed), Technische Universität Dresden, 01062 Dresden, Germany; 4 Max Planck Institute for the Physics of Complex Systems, 01187 Dresden, Germany; 5 Professur für Makromolekulare Chemie, Department Chemie, Technische Universität Dresden, 01069 Dresden, Germany; 6 Dresden Center for Computational Materials Science, TU Dresden, 01062 Dresden, Germany

## Abstract

The mechanical response of patterned graphene nanoribbons (GNRs) with a width less than 100 nm was studied *in*-*situ* using quantitative tensile testing in a transmission electron microscope (TEM). A high degree of crystallinity was confirmed for patterned nanoribbons before and after the *in*-*situ* experiment by selected area electron diffraction (SAED) patterns. However, the maximum local true strain of the nanoribbons was determined to be only about 3%. The simultaneously recorded low-loss electron energy loss spectrum (EELS) on the stretched nanoribbons did not reveal any bandgap opening. Density Functional Based Tight Binding (DFTB) simulation was conducted to predict a feasible bandgap opening as a function of width in GNRs at low strain. The bandgap of unstrained armchair graphene nanoribbons (AGNRs) vanished for a width of about 14.75 nm, and this critical width was reduced to 11.21 nm for a strain level of 2.2%. The measured low tensile failure strain may limit the practical capability of tuning the bandgap of patterned graphene nanostructures by strain engineering, and therefore, it should be considered in bandgap design for graphene-based electronic devices by strain engineering.

## Introduction

Graphene is an allotrope of carbon, and it is the first isolated two-dimensional crystalline material^1^. Graphene shows great potential in many areas due to the unique properties, particular the intriguing electronic properties^2–7^. One of the most exciting applications is in novel electronic devices, e. g., flexible electronics and high-frequency transistors^8–10^. However, the absence of a bandgap is a serious limitation for its application in transistors as a planar channel material, since switching off any kind of graphene-based field-effect transistor would be extremely difficult, not to mention the required value of ~10^6^ for the on/off ratio in logic transistors. Therefore, bandgap engineering and increasing the on/off ratio are essential for its application in electronics. Strain engineering has been proposed to open the bandgap of graphene for a few years^11–17^, since the very strong bonding between carbon atoms is supposed to offer remarkable mechanical properties of graphene. Its intrinsic strain of 25% calculated from the experimental data (Young’s modulus *E* and third-order elastic modulus *D*) in ref. 2 also seems to provide a lot of space for bandgap opening by strain engineering. However, the practical application of graphene for electronic devices (e. g. transistors) will often inevitably require patterned graphene nanostructures^18, 19^. Therefore, the investigation of strain engineering on patterned graphene-based nanostructures is a necessary step towards its practical application for future electronics.

In this study, graphene is patterned to nanoribbons with a width less than 100 nm in a transmission electron microscope (TEM) at an acceleration voltage of 200 keV, and subsequently studied and *in*-*situ* stretched in the TEM at 80 keV. The maximum local true strain of patterned graphene nanoribbons (GNRs) is determined to be about 3%, which is much less than theoretically expected. Since such a low tensile failure strain would limit the freedom of tuning the bandgap of patterned graphene nanostructures by strain engineering, it has to be considered in designing a bandgap for graphene-based electronic devices by strain engineering. Density Functional Based Tight Binding (DFTB) simulation is conducted to predict a feasible bandgap opening as a function of width in GNRs at low strain levels.

## Methods

### Sample preparation

**Figure 1:**
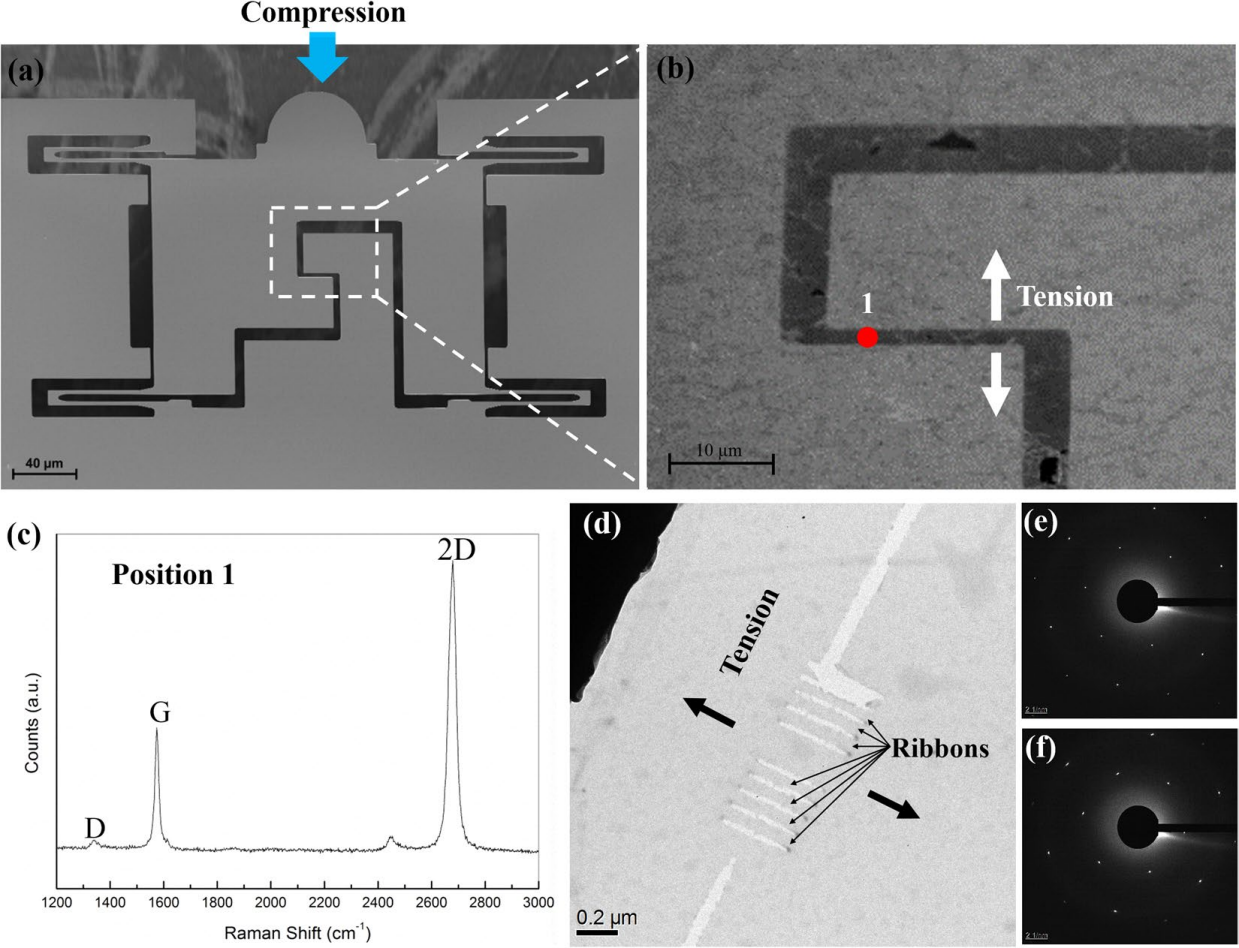
(**a**) SEM image of the PTP device, (**b**) SEM image of a successfully transferred graphene piece on the PTP device, (**c**) a typical Raman spectrum acquired from the transferred graphene on the target gap, (**d**) BF TEM image of the patterned GNRs, (**e**) SAED pattern from the graphene membrane next to the nanoribbons, and (**f**) SAED pattern from the nanoribbons.

Graphene samples were produced in a chemical vapor deposition (CVD) furnace system using copper as the catalyst and the substrate^20, 21^. The high quality of the single layer graphene after transfer to SiO_2_/Si and to the TEM lacey grid was proven by Raman spectroscopy, selected area electron diffraction (SAED) pattern and high resolution transmission electron microscopy (HRTEM) (Fig. S1–2). To transfer the graphene on the target device, a very thin film of polymethyl methacrylate (PMMA) (less than 50 nm) was spin-coated on the surface of the graphene, and subsequently, the copper was etched away using 0.1 mol/L ammonium persulfate. A floating graphene-PMMA film after copper etching was collected by a clean aluminum foil and transferred to a beaker with distilled water to rinse thoroughly. The rinse process was repeated several times to dissolve any excess ammonium persulfate. Subsequently, the graphene-PMMA film was fished up by a so-called “push-to-pull” (PTP) MEMS device (Fig. 1a). The PTP device with graphene-PMMA was heated in the oven at a temperature of 125 °C for 2 to 3 hours. Then, the PMMA was dissolved by a mixed solution (50 at% acetone and 50 at. % isopropanol alcohol (IPA)) in a critical point dryer. In the transfer process, coating a very thin PMMA layer and baking for a long time are very important to improve the adhesion between the graphene and the PTP device (Fig. S3 shows two failed transfers due to without using very thin PMMA layers and long time baking). A critical point dryer was used to eliminate the surface tension force during dissolving the PMMA layer, which could easily break the graphene on the target gap of the PTP device. A successfully transferred graphene piece on the PTP device is shown in Fig. 1b.

A typical Raman spectrum acquired from the transferred graphene on the target gap is shown in Fig. 1c. The sample was excited by a 532 nm laser (2.33 eV), the spot size of the laser beam was about 0.5 µm. The laser power was limited to 2 mW to prevent thermal heating and damage of graphene. Since there is no obvious PMMA peak at 1430 cm^−1^ visible in the Raman spectrum, it is proven that the PMMA was removed by acetone and IPA. The D peak (defect peak) at about 1345 cm^−1^ is negligible in the measured spectrum, which demonstrates the high quality of the graphene. The intensity ratio of the 2D (about 2671 cm^−1^) and G (about 1582 cm^−1^) peaks is higher than 2, which indicates that the transferred specimen is single layer graphene.

### Patterning

The graphene transferred on the PTP device was patterned into nanoribbons (Fig. 1d) by the focused electron beam in a Carl Zeiss LIBRA 200 MC Cs STEM tool at an acceleration voltage of 200 kV, using the scanning beam in STEM mode. Contrary to that normal imaging the sample requires a small spot size to select only the electrons emitted at the very tip of the source in order to achieve very high resolution, a large condenser aperture (30 µm) and high spot size (≥9) were used to carve the graphene^22^. Focused ion beam (FIB) was abandoned due to the wide lateral damage observed in our previous study^23^. A graphene ribbon (GR) patterned by FIB was severely damaged, as shown in Fig. S4. Additionally, two large cutting lines perpendicular to the GNRs were introduced in the patterning process to optimize the sample structure. This pattern geometry can avoid early fracture in the graphene membrane instead of in the GNRs. Figure S5 shows that the graphene membrane broke firstly instead of the graphene nanoribbon (GNR) due to the sample structure without two large cutting lines. The largest condenser aperture (200 μm) and large spot size (12) were used to increase the cutting speed for the two large cutting lines. Although tiny structural defects may be introduced in the patterning process, diffraction patterns from the graphene membrane and the GNRs (Fig. 1e and f) confirm their high degree of crystallinity after the patterning.

### Experimental set-up

The *in*-*situ* stretching of GNRs was performed inside a Carl Zeiss LIBRA 200 MC Cs STEM tool at an acceleration voltage of 80 kV to reduce the knock-on damage in the graphene^22, 24^, and to avoid the Cherenkov radiation in the electron energy loss spectrum (EELS)^25, 26^. A Hysitron PI 95 TEM PicoIndenter with a conductive diamond flat punch indenter was used to compress the semi-circular part of the PTP device (Fig. 1a), therefore to perform a tensile test in the middle gap (Fig. 1b)^27^. The PTP device has four identical springs with higher stiffness in the lateral direction to ensure uniaxial tensile loading. The maximum applicable displacement and load are 2 µm and 1 mN, respectively. The mechanical response of GNRs was monitored and recorded *in*-*situ* inside the TEM. Simultaneously, EELS was recorded to observe if a bandgap opening occurs by stretching the GNRs. The smallest monochromator slit was inserted in the TEM to achieve the best possible energy resolution of 0.15 eV, and to narrow the zero loss peak in the EELS spectrum.

### DFTB simulation

Electronic bandgap calculations for unstrained and strained GNRs were carried out by means of a DFTB approach using the DFTB+ code^28^. This method combines accuracy with numerical efficiency, and it allows dealing with systems up to 2000 atoms in a quantum simulation, especially for carbon based materials^29^. We have used the Slater-Koster parameters developed by Niehaus *et al.* ^30^ for C and H atoms. Cell and geometry optimizations of GNRs were performed until the absolute value of the inter-atomic forces is below 10^−5^ atomic units. Periodic boundary conditions were considered in the x-direction of the nanoribbon.

## Results and Discussion

**Figure 2:**
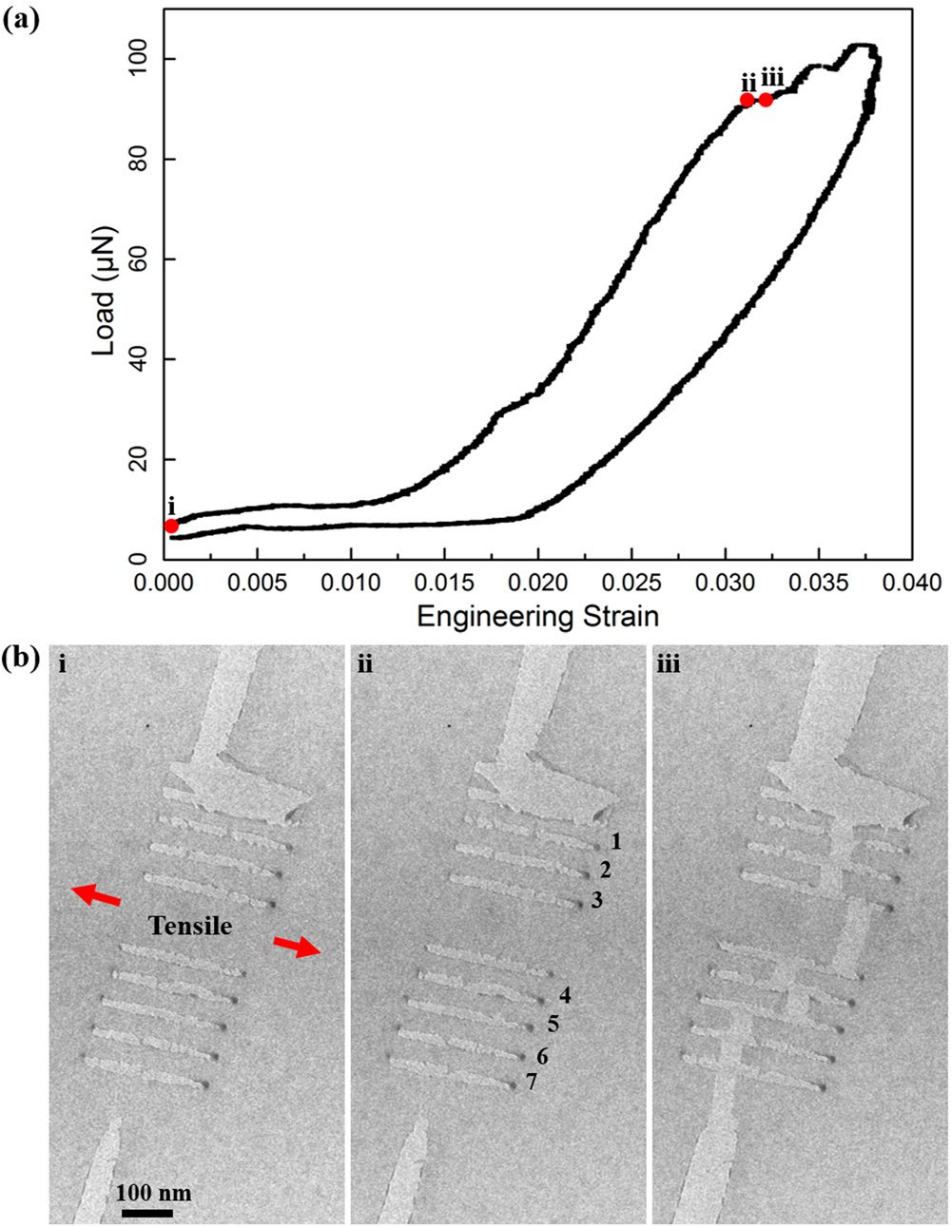
*In*-*situ* tensile experiment of GNRs. (**a**) Load-engineering strain curve (loading and unloading), and (**b**) (i) initial state, (ii) maximum strain state, and (iii) state right after the fracture.

The sample consisting of seven patterned GNRs with a width less than 100 nm (Fig. 1d) was successfully used for *in*-*situ* stretching experiment. The length of the seven GNRs was a constant value (380 nm), as shown in Table 1. SAED patterns from the graphene membrane and the GNRs (Fig. 1e and f) confirm their high degree of crystallinity before the *in*-*situ* test, and prove that the patterning by the focused electron beam in the TEM should not affect the mechanical response of GNRs to a significant amount. The force-engineering strain curve of the *in*-*situ* tensile test is shown in Fig. 2a, the engineering strain was calculated by dividing the raw displacement data by the initial gap width. The calculated engineering strain may be overestimated since the raw displacement includes not only the contribution from the gap width but also those from other parts of the PTP device. Furthermore, the true force responsible for only breaking the patterned GNRs could not be recalculated by directly introducing the spring constant of the PTP device, due to the fact that the graphene membrane along with the patterned GNRs was also stretched in the experiment. The graphene membrane was not etched away and was kept on the PTP device because (a) graphene cutting by focused electrons in the TEM is a very slow process (100–200 nm per hour) and (b) the focused electrons even with the largest condenser aperture (200 µm) and large spot size (12) in the TEM were not able to cut the folded edge (Fig. S6b) (FIB was abandoned for etching away the graphene due to the wide lateral damage (tens of µm) as discussed above^23^). Nevertheless, a strain was applied to the patterned GNRs. Representative images from the *in*-*situ* stretching test are shown in Fig. 2b. The graphene membrane and the GNRs are stretched, as shown in Fig. 2b(ii), and the corresponding engineering strain is about 3.18%. It is clearly shown that the tensile experiment was successfully performed along the longer side of the GNRs. To determine the local “true” strain applied to the GNRs, the measured strain values were recalculated based on the *in*-*situ* video information (Video S1). Digital image correlation was applied to the frames extracted from the recorded video to measure the local elongation of the GNRs. Typically, dark features on the GNRs were used as markers for the digital image correlation to avoid any diffraction contrast effects as the ribbons are deformed. Table 1 summarizes the local “true” strain of the GNRs, i. e. the strain for fracture of the GNRs, shown in Fig. 2. The breaking strain ranges from 2.8% to 3.0%, and the width of the ribbons (40–75 nm) seems to do not affect the maximum strain. The breaking “true” strain was determined to be about 3%, confirmed by more than 30 GNRs (5 samples) in the *in*-*situ* experiments. This value is much lower than the theoretically predicted one^31^ and the experimentally determined value published in ref. 2 (in both cases more than 16%). However, even the value in ref. 2 was calculated based on the determined Young’s modulus *E* and the third-order elastic modulus *D* from the experimental results. To our knowledge, our study is the first one that directly measures the tensile failure strain for patterned GNRs. The low-loss EELS was recorded to detect any change that indicates an opening of a bandgap of the stretched GNRs. The low-loss EELS measurements were performed with the highest possible energy resolution (about 0.15 eV, see Fig. S7) by inserting the smallest monochromator slit in the TEM. The energy dispersion was set to be 0.01/0.02 eV/channel to take full advantage of the energy resolution of the spectrometer. No noticeable signal of the bandgap from the stretched GNRs could be detected in the spectrum (Fig. S8). Since the tail of the zero loss peak (ZLP) extends to more than 1 eV (Fig. S8), the extended tail of the ZLP would cover the signal of a bandgap in the spectrum even if a bandgap could be opened in the stretched GNRs according to refs 12 and 32. Different ZLP subtraction methods were used to remove the ZLP of the spectrum, however, they failed to reveal any bandgap opening of the stretched GNRs (Fig. S9 and Table S1) either.

**Table 1:** The breaking local true strain of the GNRs shown in Fig. 2.

Number	1	2	3	4	5	6	7
Breaking strain (%)	2.8	2.9	2.8	2.8	2.8	3.0	3.0
Dimensions (nm*nm)	40*380	55*380	70*380	50*380	60*380	65*380	75*380

**Figure 3:**
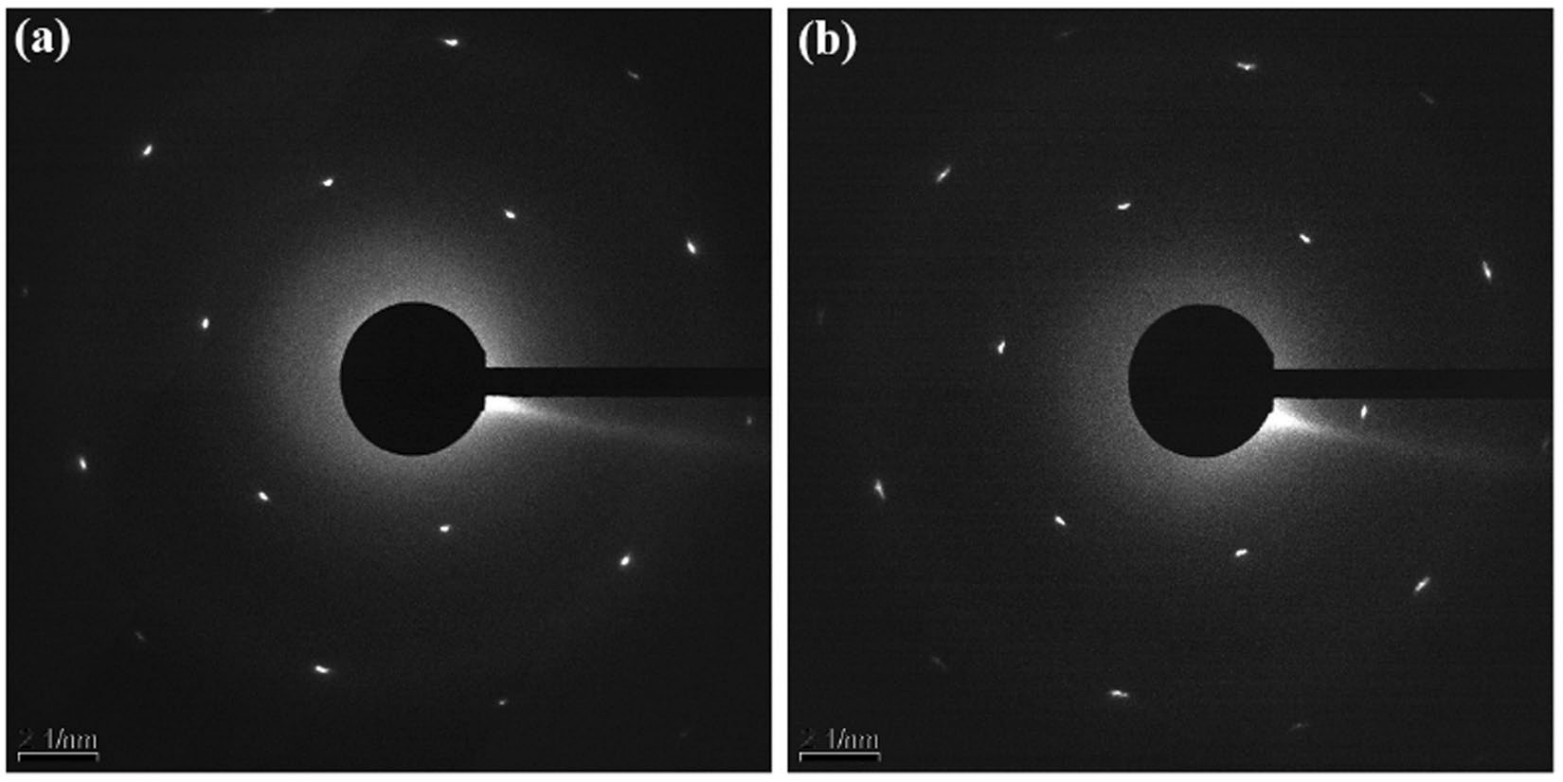
SAED patterns of the sample after the *in*-*situ* stretching experiment shown in Fig. 2. (**a**) From the graphene membrane next to the fractured GNRs, and (**b**) from the fractured GNRs.

Since the high-keV electron flux always has an effect on the *in*-*situ* experiments in the TEM, the illumination step was kept very low (≤3, maximum is 25 in the Carl Zeiss Libra 200 TEM) to minimize the effect from the electron beam on the mechanical response of GNRs in the *in*-*situ* stretching experiment. The graphene membrane was tested to be very stable even after a few hours’ illumination using the electron beam with an illumination step 4. Therefore, the illumination of less than 5 min of high-keV electron flux should not introduce significant defects into the graphene, and consequently, it should not affect the tensile strain value of GNRs during the *in*-*situ* stretching experiment in the TEM. SAED patterns of the same sample (Figs 1 and 2) after the *in*-*situ* stretching experiment are shown in Fig. 3. It indicates that the high degree of crystallinity in the membrane and in the GNRs were still preserved after the *in*-*situ* test.

One interesting phenomenon is worth to mention, i.e., that any change of magnetic lenses (changing illumination step and magnification, switching between imaging mode and spot mode, switching between imaging mode and spectrum mode, etc.) in the TEM tends to break the strained GNRs and/or graphene membrane. This behavior was typically observed when the GNRs and the graphene membrane were strained by more than 1.5%. It is speculated that the change of magnetic lenses in the used TEM generates a fluctuation of the magnetic field, resulting in a current pulse in the used capacitive force/displacement transducer (PI 95 TEM PicoIndenter), which may create a displacement jump of the conductive tip. This jump would lead to an additional strain on the sample and it would break it. Therefore, any changes of magnetic lenses during the *in*-*situ* stretching experiment should be avoided. The graphene should be at a relatively low strain stage (≤1.5%) if a change of magnetic lenses has to be performed.

**Figure 4:**
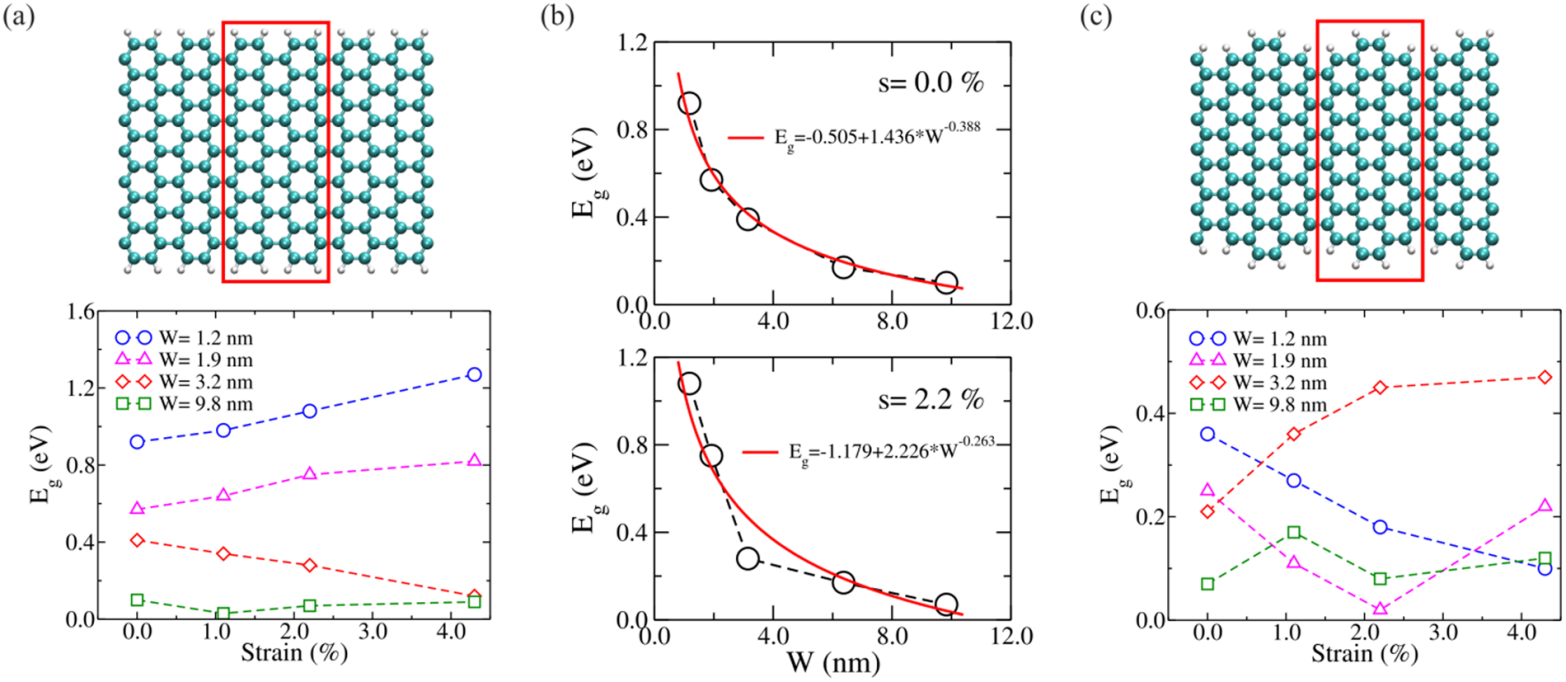
Electronic bandgap calculations of GNRs. (**a**) Strain dependence of the bandgap in armchair GNRs. (**b**) Variation of the bandgap as a function of the width for AGNRs at different strain level. (**c**) Strain dependence of bandgap for GNRs with mixed edges, armchair and zigzag. The supercell to perform the calculations is enclosed by the red rectangle in both cases.

A DFTB approach was utilized to study the electronic bandgap of unstrained and strained GNRs, which is shown in Fig. 4. The bandgap of unstrained armchair graphene nanoribbons (AGNRs) decreases with increasing width (see Fig. 4a), as expected. Under an applied strain, the bandgap tends to increase for small ribbons (e.g., up to 1.27 eV for a ribbon of W = 1.2 nm) as the strain increases, whereas the gap fluctuates for larger ribbons (e.g., the ribbon of W = 9.8 nm) and does not show a uniform trend. This result is in agreement with experimental and theoretical studies in AGNRs^33–35^. Despite this effect, we did not find any fracture of the ribbon at these small strain levels. However, by extrapolating the width dependence of the bandgap (see Fig. 4b), the bandgap of unstrained AGNRs vanishes for a width of about 14.75 nm, and the critical width reduces to 11.21 nm for a strain level of 2.2%. These data explain that for GNRs with a width of 40–75 nm as used in this experimental study, no bandgap response is detectable. The width of the GNRs used in the experiment was much bigger than the threshold value (14.95 nm without strain, 11.21 nm for a strain level of 2.2%) found in the calculation results. Therefore, the electronic bandgap of the patterned GNRs could not be opened with a maximum strain of about 3% in the experiment. As a consequence of the numerical simulations and supported by the experimental data achieved here, for the practical application (e.g., opening a reasonable bandgap > 0.7 eV at low strain level of 2.2%), graphene is required to be patterned into AGNRs with a width less than 2 nm.

In addition, the strain dependence of the bandgap for GNRs with mixed zigzag and armchair edges (see Fig. 4c) were computed. A reduction in the bandgap magnitude (E_g_-max < 0.5 eV) is detected when comparing to the results obtained for pure armchair edges, and a totally different non-monotonic fluctuating behavior of the bandgap after increasing the strain in small and large GNRs is observed. Similar to AGNRs, those ribbons did not break for strain levels up to 4.3%. It is speculated that the early breaking of the ribbons at low strain level observed in the *in*-*situ* experiment above is due to the presence of tiny structural defects, whose study goes beyond the scope of the present work. These tiny structural defects are inevitable in the producing process of CVD graphene and in the subsequent direct patterning process by the electron beam, which are also expected in the production line of future graphene electronics unless more appropriate approaches are developed.

## Conclusions

In summary, CVD graphene was transferred to a PTP device for graphene stretching by the modified PMMA transfer method, and it was successfully patterned into GNRs with widths less than 100 nm using a 200 keV focused electron beam in a TEM. The mechanical response of patterned GNRs was studied *in*-*situ* using quantitative tensile testing in the TEM at 80 kV. A high degree of crystallinity of the patterned GNRs before and after the *in*-*situ* experiment was confirmed by SAED patterns, however, the maximum strain of the ribbons was determined to be only about 3%. The recorded low-loss EELS on the stretched ribbons did not reveal any bandgap opening for the studied GNRs. DFTB simulation was conducted to predict a feasible bandgap opening as a function of width in GNRs with low strain. The bandgap of unstrained AGNRs vanished for a width of about 14.75 nm, and this critical width reduced to 11.21 nm for a strain level of 2.2%. These data explain that no bandgap opening in strained nanoribbons with a width of 40–75 nm is detectable. In order to open a reasonable bandgap > 0.7 eV at a strain level of 2.2%, graphene should be patterned into AGNRs with a width less than 2 nm. The measured low tensile failure strain may limit the practical capability of tuning the bandgap of patterned graphene nanostructures by strain engineering, and should be considered in the bandgap design for graphene-based electronic devices by strain engineering.

## Electronic supplementary material

Supplementary information

Video S1
