# Supplementary material for: In-Situ Stretching Patterned Graphene Nanoribbons in the Transmission Electron Microscope
Source: Sci Rep. 2017 Mar 16;7:211. doi: 10.1038/s41598-017-00227-3 (PMC5428052; doi:10.1038/s41598-017-00227-3)
Supplement: Supplementary file 1 — Supplementary information [file 41598_2017_227_MOESM1_ESM.docx]

**Supplementary Information**

**In-Situ Stretching Patterned Graphene Nanoribbons in the Transmission Electron Microscope**

Zhongquan Liao ^1,2,3^*, Leonardo Medrano Sandonas ^2,4^, Tao Zhang ^5^, Martin Gall ^1^, Arezoo Dianat ^2^, Rafael Gutierrez ^2^, Uwe Mühle ^1^, Jürgen Gluch ^1^, Rainer Jordan ^5^, Gianaurelio Cuniberti ^2,3,6^, and Ehrenfried Zschech ^1,3^

^1^ Fraunhofer Institute for Ceramic Technologies and Systems (IKTS), 01109 Dresden, Germany

^2^ Institute for Materials Science and Max Bergmann Center of Biomaterials, Technische Universität Dresden, 01069 Dresden, Germany

^3^ Center for Advancing Electronics Dresden (cfaed), Technische Universität Dresden, 01062 Dresden, Germany

^4^ Max Planck Institute for the Physics of Complex Systems, 01187 Dresden, Germany

^5^ Professur für Makromolekulare Chemie, Department Chemie, Technische Universität Dresden, 01069 Dresden, Germany

^6^ Dresden Center for Computational Materials Science, TU Dresden, 01062 Dresden, Germany

^*^ [Zhongquan.liao@ikts.fraunhofer.de](mailto:Zhongquan.liao@ikts.fraunhofer.de)


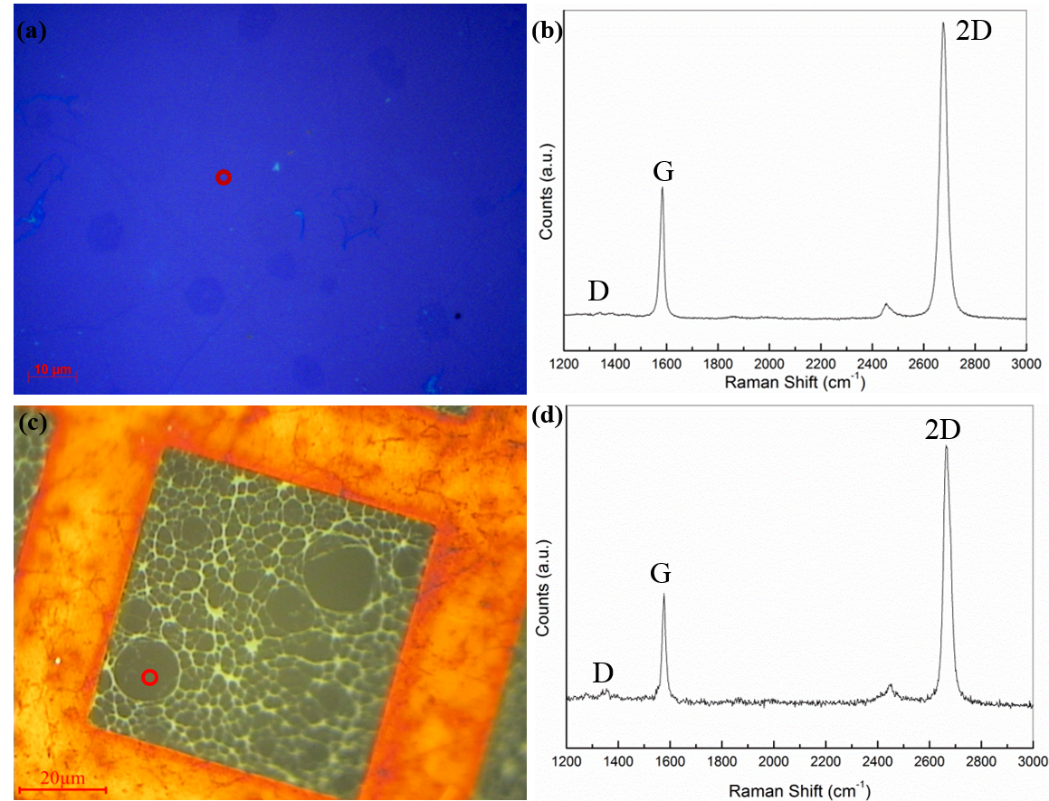


**Figure S1.** (a) and (c) Optical microscopy images of the transferred CVD graphene on different substrates (SiO_2_/Si in (a) and TEM lacey grid in (c)), and (b) and (d) the corresponding Raman spectra.


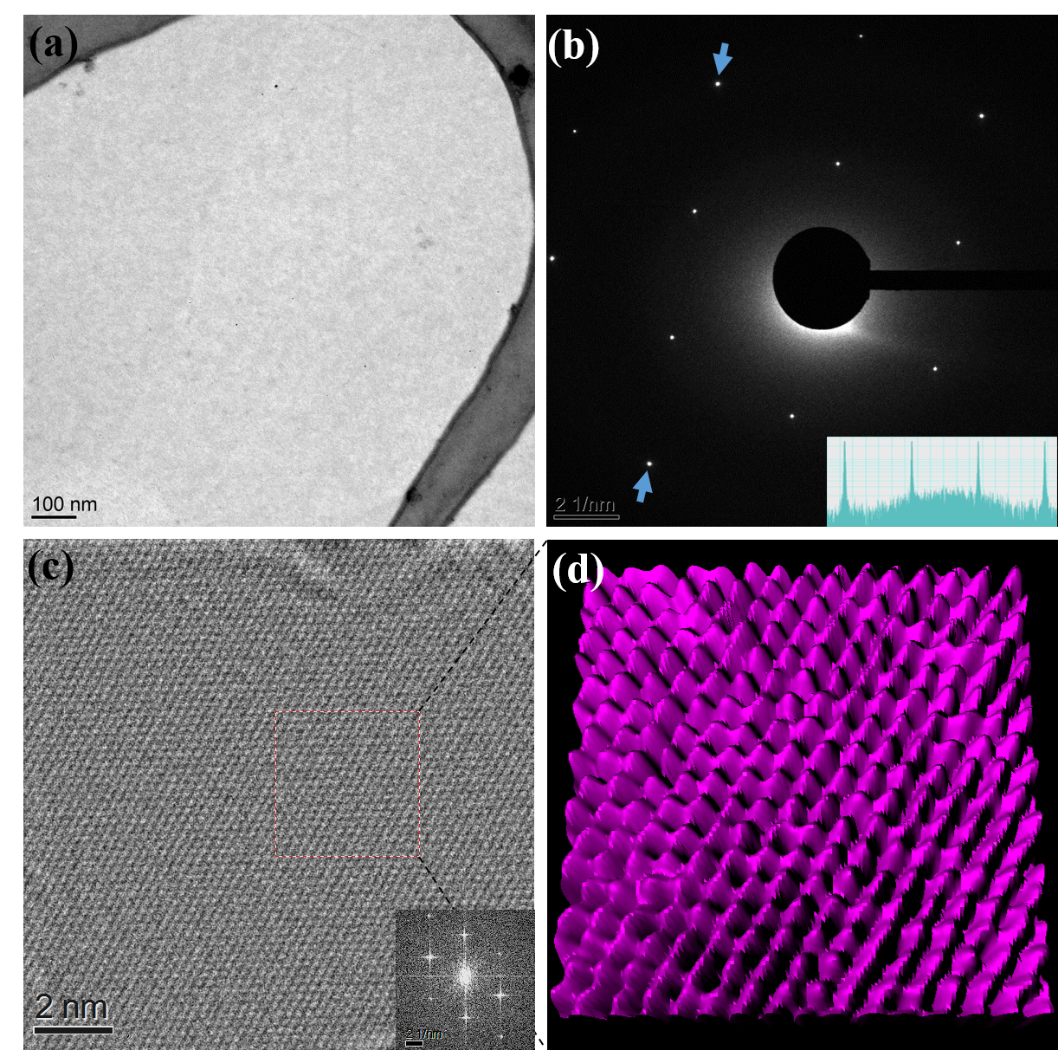


**Figure S2**. TEM study on a single layer CVD graphene transferred to a lacey TEM grid. (a) Low-magnification TEM image; (b) the corresponding diffraction pattern, and the intensity profile plots taken between the blue arrows (inset); (c) high resolution TEM image, and the corresponding fast Fourier transform pattern (inset); and (d) a false color 3D image originated from the Fourier enhanced TEM micrograph of the marked area in (c).


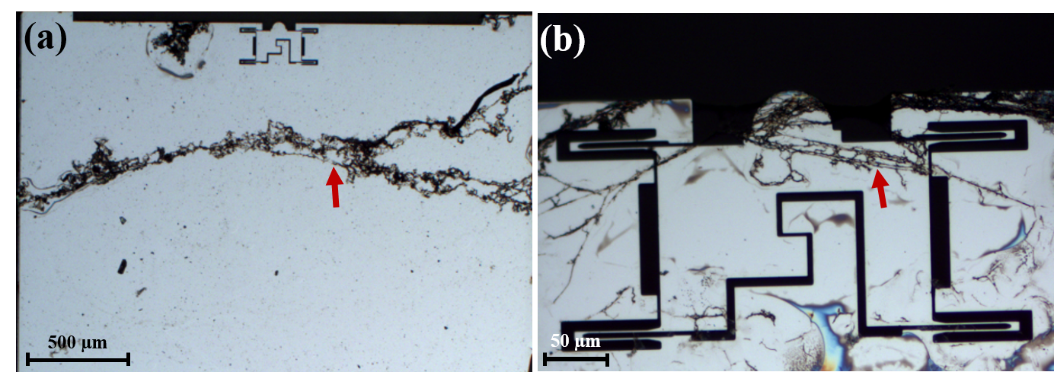


**Figure S3.** Optical microscopy images of two PTP devices with unsuccessfully transferred graphene (the entangled graphene crap is shown by red arrows).


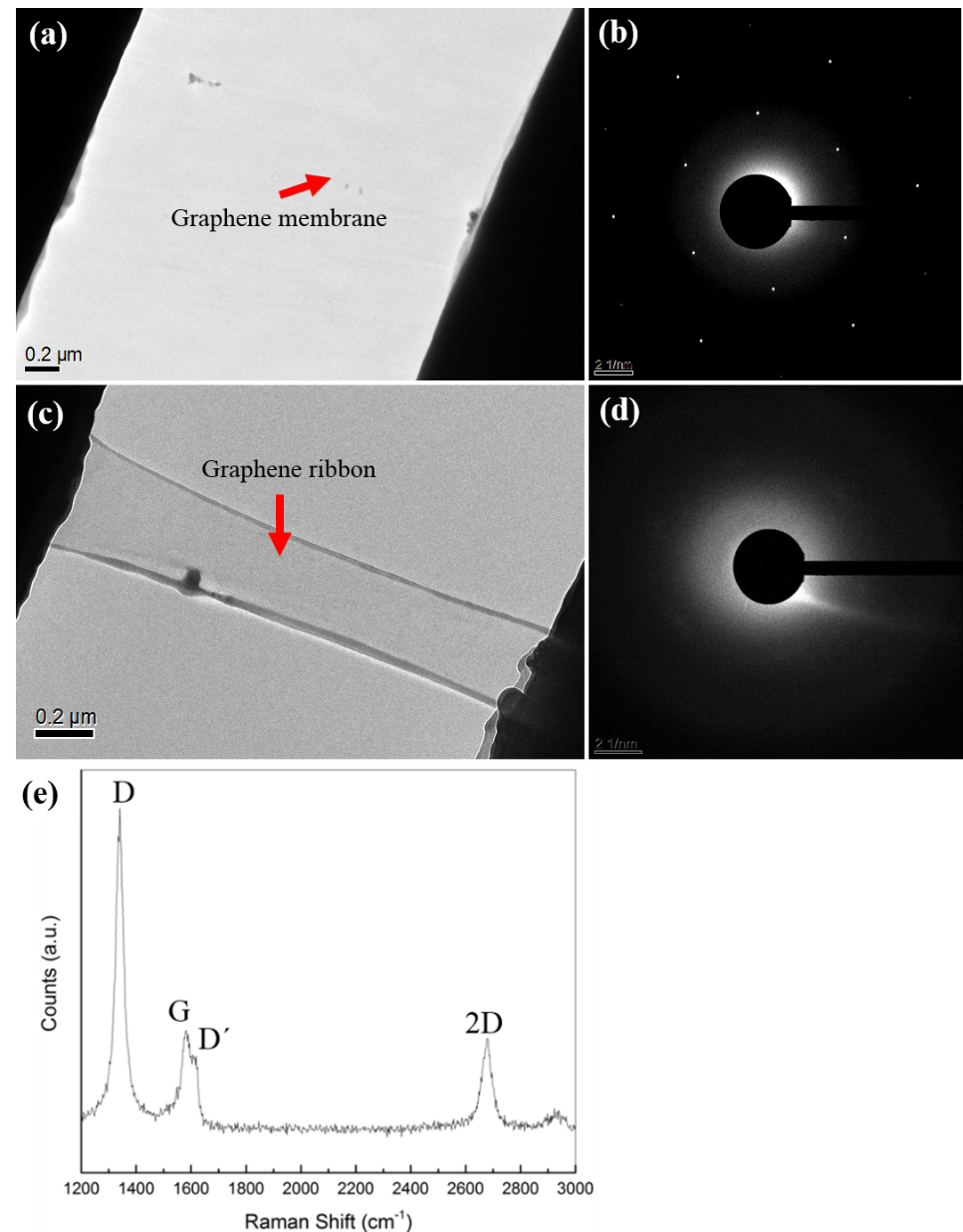


**Figure S4.** (a) BF TEM image of a monolayer graphene membrane on the 2.5 µm wide target gap of a PTP device; (b) the SAED pattern of the graphene membrane shown in (a); (c) BF TEM image of a monolayer GR on the 2.5 µm wide target gap of the PTP device, which was patterned by using a Ga^+^ ion beam (30 kV, 1 pA/10 s for both sides, the GR was not irradiated by the ion beam in the whole patterning experiment); (d) the SAED pattern from the patterned GR shown in (c); and (e) Raman spectrum from the patterned GR shown in (c).


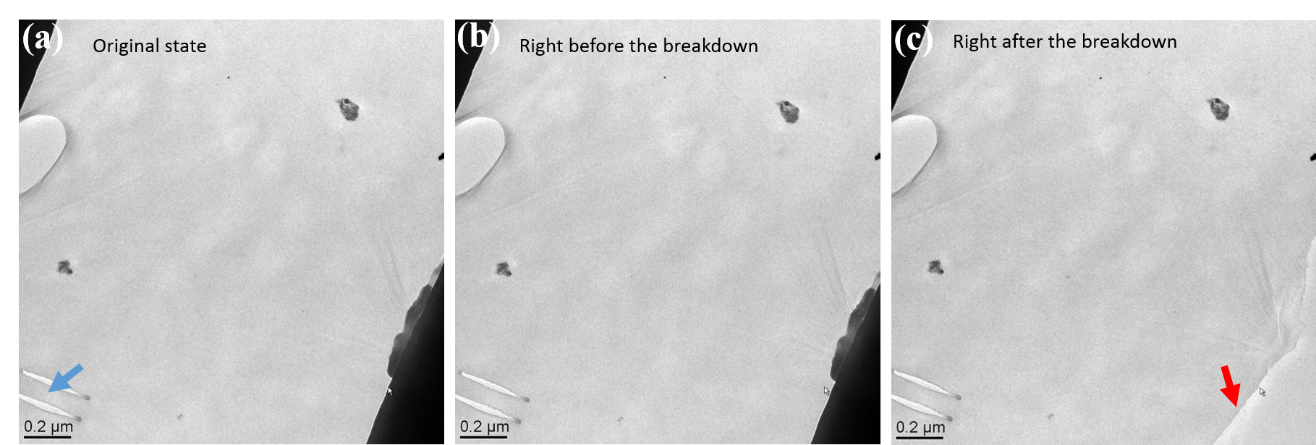


**Figure S5.** Representative TEM images of a GNR patterned by focused electron beam in the in-situ stretching experiment. (a) At the original state, (b) at the maximum strain state, and (c) at the state right after the breakdown. The GNR is indicated by a blue arrow in (a). The graphene membrane breaks earlier than the GNR due to not optimized patterning geometry in this special case.


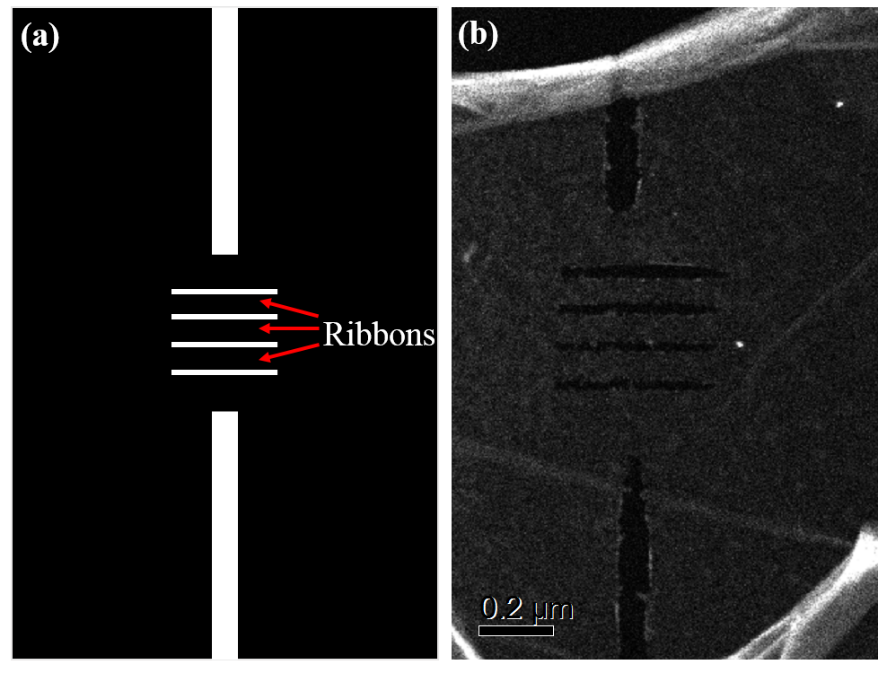


**Figure S6.** Optimized patterning geometry of the experimental sample. (a) Schematic picture, and (b) high angle annular dark field (HAADF) STEM image of a patterned sample on the PTP device by focused electrons. Two large cutting lines were introduced in the optimized patterning geometry to ensure the GNRs break firstly.


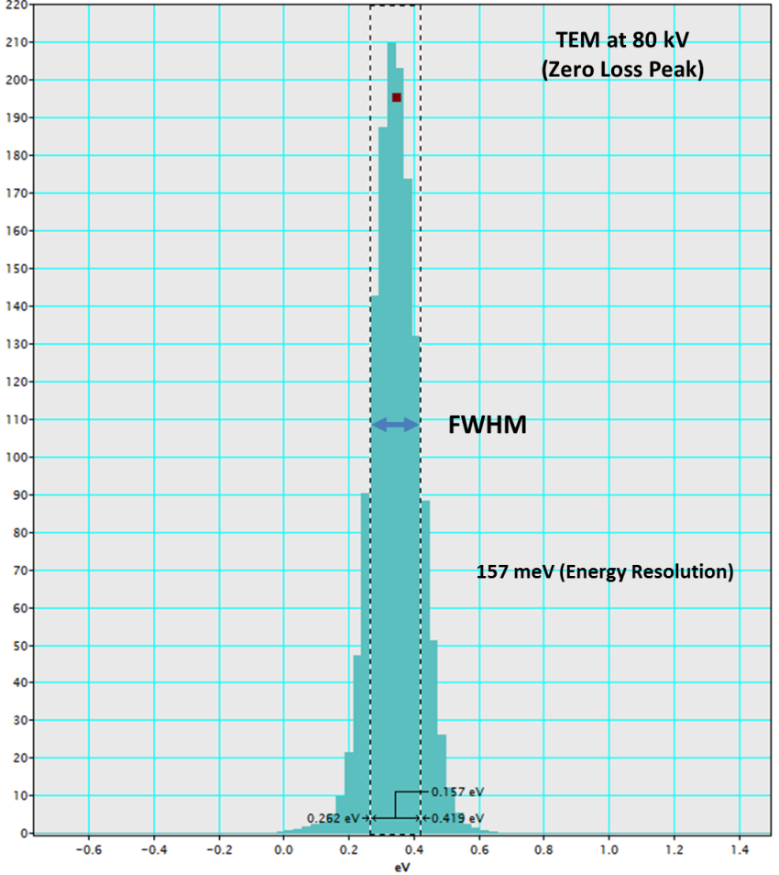


**Figure S7.** The measured electron energy spread at 80 kV with a smallest monochromator slit acquired for 1 s.





**Figure S8.** Low-loss EELS of the GNRs with and without strain for a tested sample. Although a very high energy resolution (about 0.15 eV) is achieved, the tail of the ZLP extends to more than 1 eV. No noticeable signal of the bandgap from the stretched GNRs could be directly detected in the spectrum.


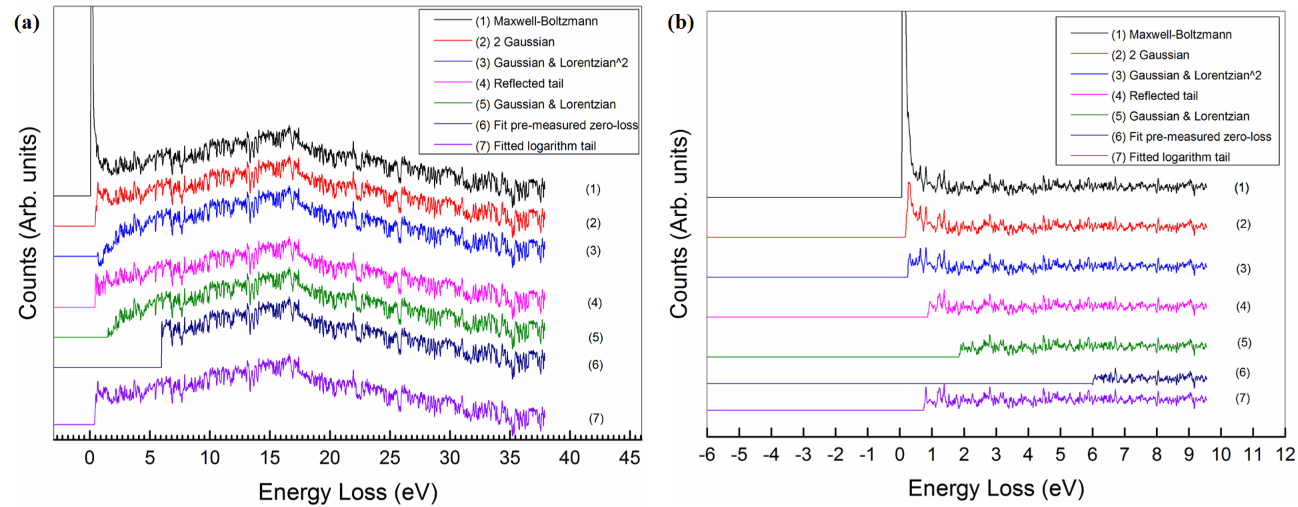


**Figure S9.** Comparison in several ZLP subtraction methods. EELS after ZLP subtraction for GNRs (a) without strain, and (b) at 1.25 % strain. Unfortunately, no data sets of the onset number from unstrained and strained GNRs can be used to extract bandgap data.

**Table S1.** The onset of the low-loss EELS after ZLP subtraction with several methods (summarized from Fig. S9).

| ZLP subtraction method | Maxwell-Boltzmann | 2 Gaussian | Gaussian & Lorentzian^2 | Reflected tail | Gaussian & Lorentzian | Fit pre-measured zero-loss | Fitted logarithm tail |
| --- | --- | --- | --- | --- | --- | --- | --- |
| No strain (eV) | 0.08 | 0.44 | 0.62 | 0.44 | 1.50 | 5.98 | 0.40 |
| 1.25 % strain (eV) | 0.07 | 0.18 | 0.25 | 0.87 | 1.84 | 5.99 | 0.74 |

**Video S1.** The movie from the in-situ stretching experiment in the TEM.
